# Long-term trends of nasopharyngeal carcinoma mortality in China from 2006 to 2020 by region and sex: an age-period-cohort analysis

**DOI:** 10.1186/s12889-023-16892-1

**Published:** 2023-10-20

**Authors:** Xinru Guo, Jiameng Cui, Xin Yuan, Zibo Gao, Ge Yu, Hao Wu, Changgui Kou

**Affiliations:** 1 Department of Epidemiology and Biostatistics, School of Public Health, Jilin University, No. 1163 Xinmin Street, Changchun, 130021 China; 2 Department of Social Medicine and Health Management, School of Public Health, Jilin University, No. 1163 Xinmin Street, Changchun, 130021 China

**Keywords:** Age-period-cohort model, China, Joinpoint model, Mortality, Nasopharyngeal carcinoma

## Abstract

**Background:**

China has a high mortality from nasopharyngeal carcinoma (NPC). The NPC mortality trends in China from 2006 to 2020 were described and analyzed to understand its epidemiological characteristics by region and sex and to explore age, period, and cohort effects.

**Methods:**

This study utilized NPC mortality data from the China Health Statistical Yearbook. A joinpoint regression model was used to fit the standardized NPC mortality and age-specific mortality. The age-period-cohort model was applied to investigate age, period, and cohort effects on NPC mortality risk.

**Results:**

The results showed that the NPC mortality rate in China has been declining steadily. From 2006 to 2020, the standardized NPC mortality rate in most age groups showed a significant downward trend. The annual percentage change was smaller in rural areas than in urban areas. The mortality risks of rural males and rural females from 2016 to 2020 were 1.139 times and 1.080 times those from 2011 to 2015, respectively. Both urban males born in 1984–1988 and rural males born in 1979–1983 exhibited an increasing trend in NPC mortality risk.

**Conclusions:**

Our study confirmed the effectiveness of NPC prevention and treatment strategies in China from 2006 to 2020. However, it underscored the urgent need for targeted interventions in rural areas to further reduce NPC mortality rates.

**Supplementary Information:**

The online version contains supplementary material available at 10.1186/s12889-023-16892-1.

## Background

Nasopharyngeal carcinoma (NPC) is a rare malignancy that originates from nasopharyngeal epithelial cells [1]. In 2020, a total of 133,354 new cases and 80,008 deaths from NPC were reported, mainly in East and Southeast Asia [2]. In China, the NPC incidence and mortality have become the highest among patients with otorhinolaryngologic tumors [3]. NPC demonstrates a notable prevalence and mortality burden in China, securing global rankings of 16th and 32nd, respectively [4]. The incidence and mortality of NPC in China are primarily concentrated in the southern and eastern regions, notably in five provinces: Guangdong, Hainan, Guangxi, Hunan, and Fujian. In contrast, the occurrence is relatively sparse in the northern and western areas [5]. The 5-year relative survival rate of NPC in China is low, at only 43.8% [6]. In general, the incidence of NPC in China is relatively high, and with population aging and the increase in risk factors, it may continue to rise. Furthermore, NPC has a poor prognosis and high mortality rate. Therefore, there is a heavy mortality burden from NPC in China.

In recent years, age-period-cohort studies on the incidence and mortality rates of NPC in China have also corroborated this trend. However, these studies have often focused on specific regions [7, 8], and the data have frequently been sourced from the Global Burden of Disease study [9, 10], which does not differentiate between urban and rural areas. Meng’s research [11] demonstrated a significant association between NPC survival rates and socioeconomic status. The growing disparities in urban and rural development in China have been increasingly scrutinized, with health disparities standing out as one of the crucial issues in need of urgent resolution. To contribute to addressing this pressing public health concern, we studied the time trends and age-period-cohort effects on NPC mortality by region and sex in China from 2006 to 2020.

## Methods

### Data source

The NPC mortality data used in this study were obtained from the 2007–2021 China Health Statistical Yearbook, which collects statistical data on the health of residents across 31 provinces, autonomous regions, and municipalities in China and uses the ICD-10 International Classification of Diseases statistical standard. The population data of the corresponding years came from the China Population & Employment Statistical Yearbook. The mortality data were classified by the death causes monitoring system, which is managed by the Health Information and Statistics Center of the National Health Commission, which conducts a nationally representative death monitoring project. The monitored population has increased to 24% of the Chinese population, and the number of monitoring points has increased to 605. Each monitoring point covers one district or county. Therefore, the data are well representative urban and rural areas throughout the country. In this study, the 2006–2020 NPC mortality data were statistically analyzed by region and sex (urban male, urban female, rural male, and rural female). Considering that in actual clinical practice, the number of deaths from NPC is low among people under the age of 30, and the cause of death is often complicated for people over 80 years old, this study conducted a statistical analysis of the 30-79-year-old population in China. We divided the population into ten age groups, each spanning five years (30–34, 35–39, …, 75–79) and applied five-year intervals to period groups (2006–2010, 2011–2015, 2016–2020). To prevent data overlap between adjacent cohorts, we substituted the years 2008, 2013, and 2018 for the three period groups in the age-period-cohort model. According to the linear relationship among birth cohort, period, and age (period = age + cohort), the birth cohort was divided into twelve groups (1929–1933, 1934–1938, …, 1984–1988). Using data from the seventh national census in 2020 as a reference, the direct standardization method was used to calculate the age-standardized mortality rates from 2006 to 2020.

### Joinpoint regression model

In this study, JoinPoint Regression 4.9.0.0 was used to fit the joinpoint regression model for the NPC mortality trends and calculate the annual percentage change (APC) and the average annual percentage change (AAPC). APC is a method for characterizing how cancer rates change over time, while AAPC serves as a consolidated measure summarizing trends within a specified fixed interval. The number of turning points was determined by the weighted Bayesian information criterion. If there is no turning point in the graph, then the trend is flat. APC > 0 indicates that the mortality rate increases each year during the observation period; otherwise, it decreases. The inspection level α was 0.05.

### Age-period-cohort model

The age-period-cohort model was implemented using Stata 17.0 (StataCorp, College Station TX, USA). This statistical model is based on the Poisson distribution. By controlling for the interplay among age, period, and cohort, the age-period-cohort model can provide a clearer understanding of the disease trends across age groups, time periods, and birth cohorts. Consequently, in recent years, this model has been widely employed to investigate the epidemiological characteristics of malignant tumors [12, 13]. Given the complete linear dependence among age, period, and cohort, a unique solution cannot be obtained. To solve this problem, Fu [14] and Yang et al. [15] proposed the intrinsic estimator (IE) method to provide unbiased and relatively efficient parameter estimation results. Therefore, our study employed age-period-cohort analysis in conjunction with the IE method.

## Results

### Descriptive analysis of the mortality rate

**Fig. 1:**
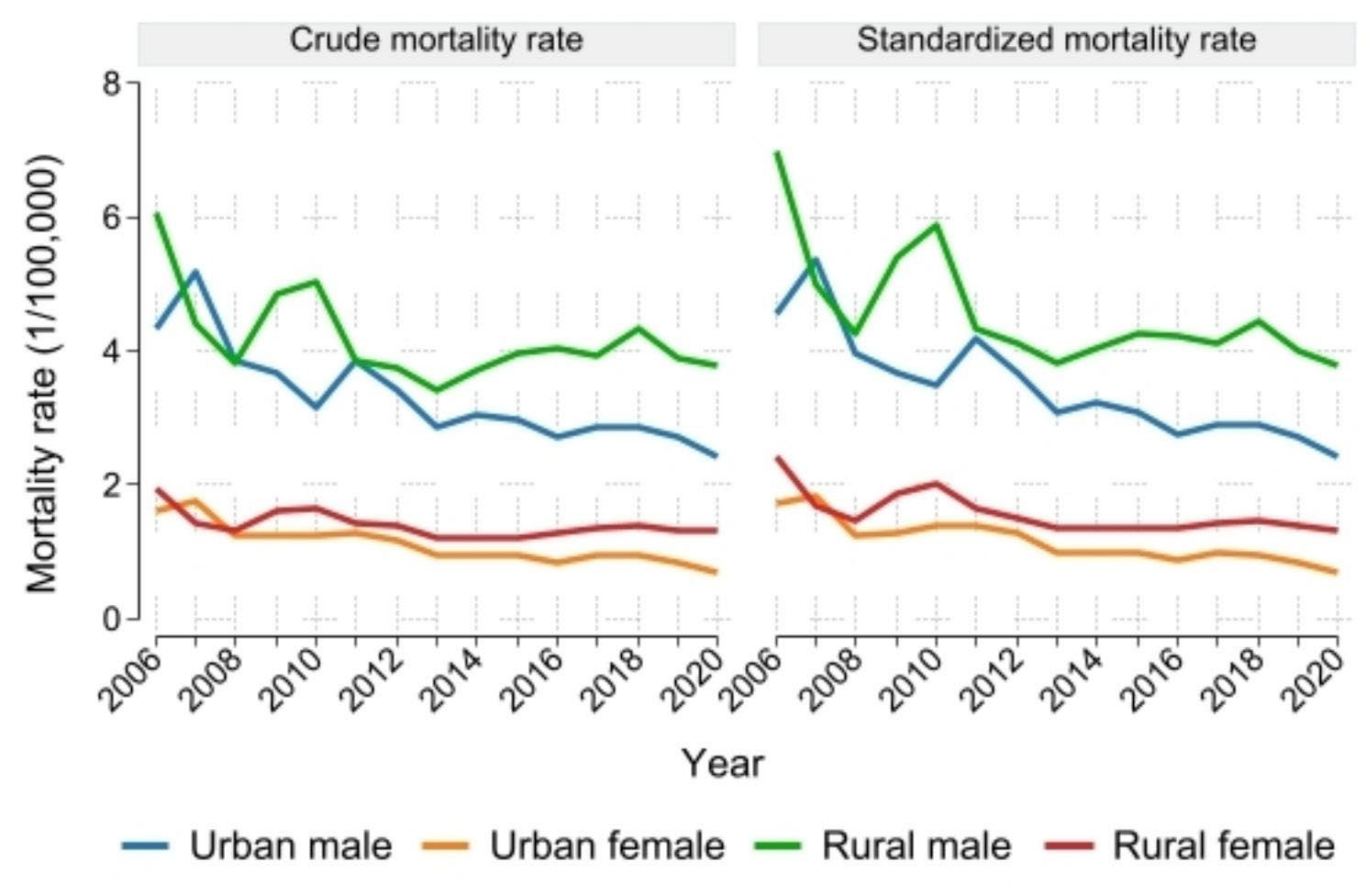
Changes in the crude and standardized mortality rates of NPC by region and sex in China (1/100,000)

Overall (Fig. 1), the crude mortality rate and the standardized mortality rate of NPC in each group showed a continuous decline from 2006 to 2020, with obvious differences between urban and rural areas and between males and females. The mortality rate was generally higher in rural areas than in urban areas and higher in males than in females, with the sex difference being more pronounced. The relevant data values are provided in Supplementary **Table A. 1**.

From 2006 to 2020, the standardized mortality rate of NPC in each group showed a significant downward trend (Table 1), with no turning point identified in any group. The APCs of urban males, urban females, rural males, and rural females were -4.5%, -5.6%, -3.0%, and -3.1%, respectively. The most substantial decline in NPC was observed in urban females, and the decline in urban areas was generally higher than that in rural areas.

**Table 1:** APC and AAPC of the standardized mortality rate of NPC

	Years	APC (%)	95%CI	AAPC (%)	95%CI
Urban male	2006–2020	-4.5*	(-5.6, -3.4)	-4.5*	(-5.6, -3.4)
Urban female	2006–2020	-5.6*	(-6.8, -4.3)	-5.6*	(-6.8, -4.3)
Rural male	2006–2020	-3.0*	(-4.6, -1.3)	-3.0*	(-4.6, -1.3)
Rural female	2006–2020	-3.1*	(-4.6, -1.6)	-3.1*	(-4.6, -1.6)

APC: annual percentage change; AAPC: average annual percentage change; CI: confidence interval

* Significantly different from 0 at alpha = 0.05 (P < 0.05)

### Trends in the age-specific mortality rate using joinpoint regression analysis

Urban males and urban females showed an overall downward trend in all age groups (Table 2). From 2006 to 2020, urban males aged 35–39 demonstrated the most pronounced downward trend (APC = -7.5%, P < 0.05), while urban females aged 40–44 exhibited the most substantial decrease (APC = -8.1%, P < 0.05). In rural areas, NPC mortality among males aged 30–34 increased at a rate of 2.9%, though most age groups showed a downward trend (ages 40–44, 45–49, 50–54, 55–59, 60–64, 65–69, 70–74, and 75–79), all with statistical significance. Among them, the age group 40–44 demonstrated the most significant decline, with an AAPC of -6.7% (P < 0.05). Among rural females, statistical significance was observed in the age groups 30–34, 35–39, 40–44, 55–59, 60–64, 65–69, and 70–74, all of which displayed a decreasing trend. Notably, the age group 40–44 exhibited the most significant decline (APC = -5.8%, P < 0.05).

**Table 2:** AAPC and APC of the age-specific mortality of NPC

Age	Urban male/Rural male			Urban female/Rural female		
	Period	APC(%, 95%CI)	AAPC(%, 95%CI)	Period	APC (%, 95%CI)	AAPC(%, 95%CI)
**30–34**	2006–2020	-1.1 (-5.1, 3.1)	-1.1 (-5.1, 3.1)	2006–2020	-3.7 (-9.1, 2.1)	-3.7* (-9.1, 2.1)
35–39	2006–2020	-7.5* (-11.2, -3.7)	-7.5* (-11.2, -3.7)	2006–2020	-7.9*(-11.9, -3.7)	-7.9* (-11.9, -3.7)
40–44	2006–2020	-5.4* (-7.8, -2.9)	-5.4* (-7.8, -2.9)	2006–2020	-8.1* (-9.9, -6.2)	-8.1* (-9.9, -6.2)
45–49	2006–2020	-6.8* (-8.8, -4.7)	-6.8* (-8.8, -4.7)	2006–2020	-7.4* (-9.7, -4.9)	-7.4* (-9.7, -4.9)
50–54	2006–2020	-4.8* (-6.9, -2.5)	-4.8* (-6.9, -2.5)	2006–2020	-5.6* (-8.3, -2.8)	-5.6* (-8.3, -2.8)
55–59	2006–2020	-5.4* (-7.5, -3.3)	-5.4* (-7.5, -3.3)	2006–2020	-6.0* (-8.3, -3.6)	-6.0* (-8.3, -3.6)
60–64	2006–2020	-3.6* (-5.2, -2)	-3.6* (-5.2, -2)	2006–2020	-4.8* (-6.9, -2.6)	-4.8* (-6.9, -2.6)
65–69	2006–2020	-2.5* (-3.9, -0.9)	-2.5* (-3.9, -0.9)	2006–2020	-5.3* (-6.2, -4.3)	-5.3* (-6.2, -4.3)
70–74	2006–2020	-3.4* (-5.1, -1.6)	-3.4* (-5.1, -1.6)	2006–2020	-4.3* (-6.4, -2.2)	-4.3* (-6.4, -2.2)
75–79	2006–2020		-4.8* (-10.5, 1.2)	2006–2020	-4.6* (-6.4, -2.7)	-4.6* (-6.4, -2.7)
	2006–2008	-20.3 (-42.3, 10.2)				
	2008–2018	-5.3* (-8.7, -1.9)				
	2018–2020	16.6 (-18.9, 67.6)				
**30–34**	2006–2020	2.9 (-1.3, 7.3)	2.9* (-1.3, 7.3)	2006–2020	-1.1 (-4.4, 2.3)	-1.1* (-4.4, 2.3)
35–39	2006–2020	0.0 (-3.0, 3.1)	0.0 (-3.0, 3.1)	2006–2020	-3.6* (-6.9, -0.3)	-3.6* (-6.9, -0.3)
40–44	2006–2020		-6.7* (-12.9, 0)	2006–2020	-5.8* (-8.6, -3)	-5.8* (-8.6, -3)
	2006–2008	-25.5 (-54.2, 21.1)				
	2008–2020	-3.1 (-7.1, 1)				
45–49	2006–2020	-1.9 (-4.8, 1.1)	-1.9* (-4.8, 1.1)	2006–2020		0.5 (-6.2, 7.6)
				2006–2009	32.2 (-6.2, 86.4)	
				2009–2020	-6.8* (-9.9, -3.5)	
50–54	2006–2020		-4.6* (-9.5, 0.5)	2006–2020		-2.3 (-9.1, 5)
	2006–2011	-10.3* (-16.2, -3.9)		2006–2008	-23.8 (-55, 28.9)	
	2011–2018	5.2 (-0.8, 11.5)		2008–2020	1.8 (-2, 5.8)	
	2018–2020	-21.1 (-44.4, 12)				
55–59	2006–2020		-4.1* (-6.9, -1.2)	2006–2020		-5.7* (-10.1, -1.1)
	2006–2017	-7.9* (-9.5, -6.3)		2006–2009	-19.2* (-34.6, -0.2)	
	2017–2020	11.3 (-3.5, 28.5)		2009–2020	-1.6 (-5.3, 2.2)	
60–64	2006–2020	-3.2* (-5.0, -1.2)	-3.2* (-5.0, -1.2)	2006–2020	-4.8* (-7.7, -1.9)	-4.8* (-7.7, -1.9)
65–69	2006–2020	-2.5 (-5.3, 0.5)	-2.5* (-5.3, 0.5)	2006–2020	-3.0 (-6.0, 0.0)	-3.0* (-6.0, 0.0)
70–74	2006–2020	-2.9* (-5.1, -0.7)	-2.9* (-5.1, -0.7)	2006–2020	-3.7* (-6.3, -1.1)	-3.7* (-6.3, -1.1)
75–79	2006–2020		-4.0* (-8.4, 0.6)	2006–2020		-1.1 (-7.9, 6.1)
	2006–2008	-18.6 (-42.6, 15.3)		2006–2010	3.6 (-6.4, 14.6)	
	2008–2020	-1.3 (-3.5, 0.8)		2010–2013	-15.4 (-40.9, 21.1)	
				2013–2020	2.9 (-1.9, 7.9)	

APC: annual percentage change; AAPC: average annual percentage change; CI: confidence interval

*Significantly different from 0 at alpha = 0.05 (P < 0.05)

### Effect of variations in age, period, and cohort on the mortality rate

**Fig. 2:**
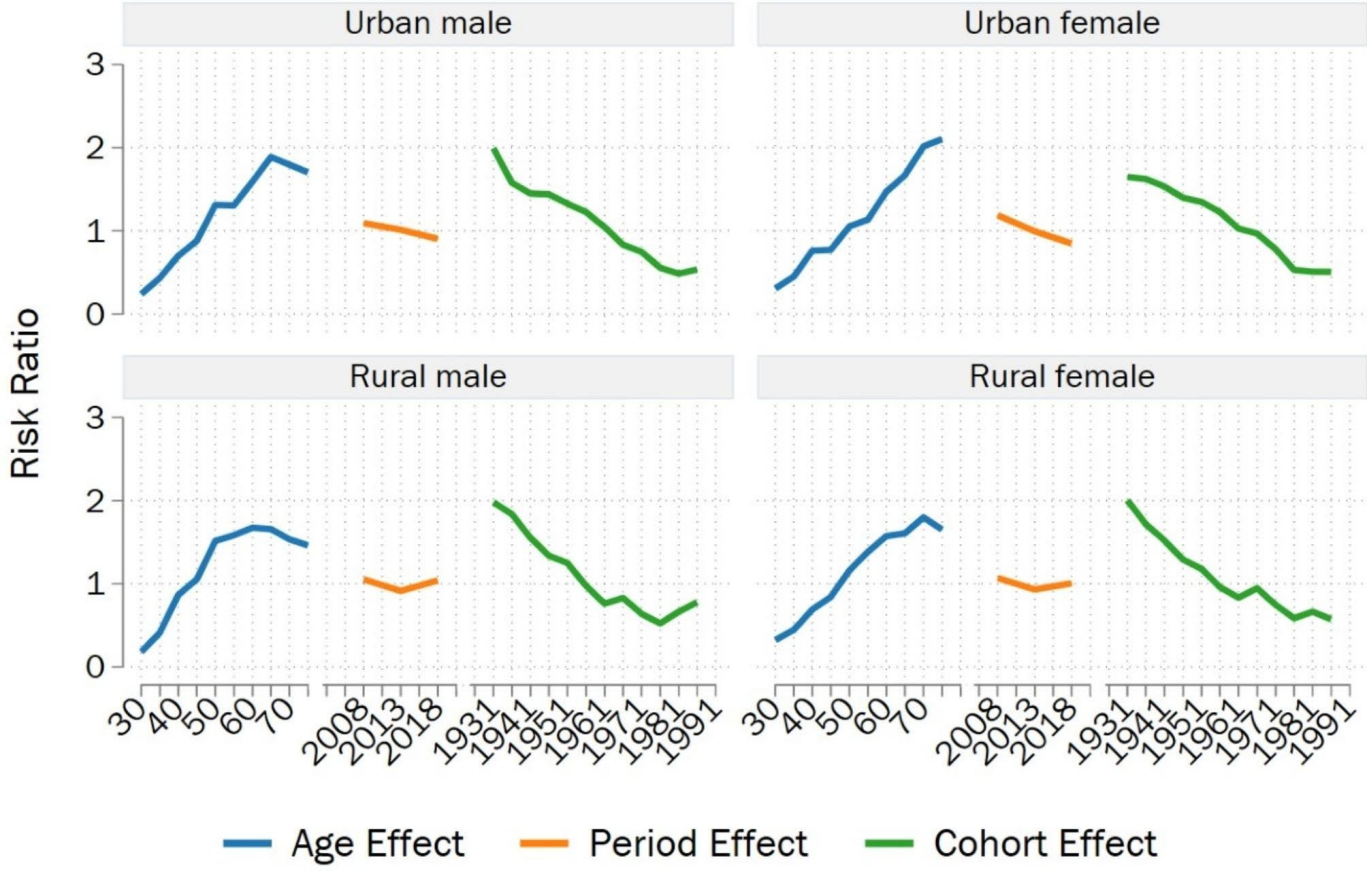
Risk Ratio of the NPC mortality of age, period, and cohort factors

Overall, the age effect coefficients of NPC mortality risk in the four groups increased with age (**Table A. 2**). The peak age effect coefficients were observed in the age group 65–69, 75–79, 60–64, and 70–74, for urban males, urban females, rural males, and rural females, respectively. These coefficients increased by factors of 2.067, 1.928, 2.235, and 1.722 compared to the coefficient in the age group 30–34, corresponding to a mortality risk increase of 7.901, 6.876, 9.346, and 5.596 times, respectively. With the advancement of the time period, the period effect coefficients of NPC mortality risk in urban males and urban females decreased, whereas they initially decreased and then increased in rural males and rural females. The effect coefficients of urban males and urban females reached their lowest points in the period from 2016 to 2020, with the risk of death in 2006–2010 being 1.208 times and 1.402 times that in 2016–2020, respectively. In rural males and females, the period effect coefficients were lowest in 2011–2015, and the mortality risk in 2016–2020 was 1.139 times and 1.080 times that in 2011–2015, respectively. Regarding cohort effects on NPC mortality risk in the four groups, an overall decreasing trend was observed. The cohort effect coefficient was highest for individuals born in 1929–1933 and lowest for urban males, urban females, rural males, and rural females born in 1979–1983, 1984–1988, 1974–1978, and 1984–1988, respectively. From the overall comparison of the three effects, the span of the age effect was the largest. Notably, NPC mortality risk increased for urban males born in 1984–1988 and rural males born in 1979–1983. For later-born cohorts, the decline in NPC mortality risk slowed for urban females, while it exhibited fluctuations for rural females (Fig. 2).

## Discussion

To the best of our knowledge, this is the first investigation in which NPC mortality data have been grouped by region and sex to compare and describe time trends and age-period-cohort effects. As a common head and neck cancer, NPC exhibits distinct geographical distribution and familial aggregation [16, 17]. The etiology of NPC remains incompletely understood, likely arising from intricate interactions among various risk factors. These factors include Epstein-Barr virus (EBV) infection [18, 19], genetic susceptibility, and environmental factors. Notably, EBV-DNA is regularly associated with the occurrence and development of undifferentiated NPC [20]. Smoking has been identified as a significant risk factor for NPC [21]. The consumption of salted fish and other preserved foods has been closely related to the risk of NPC, particularly prevalent in regions with a high incidence of NPC [22]. Additional risk factors encompass exposure to fumes, smoke, dust, and chemicals [23]. Furthermore, evidence suggests that a diet rich in fresh fruits and vegetables plays a protective role against NPC development [24, 25].

In this study, the NPC mortality rate in males was much higher than that in females, which is consistent with previous studies [10, 26]. The higher NPC mortality rate in rural areas, compared to urban areas, may be attributed not only to disparities in medical infrastructure but also to lifestyle factors. This discrepancy might be associated with the prevalence of unhealthy working conditions and increased exposure to smoke and chemicals among migrant workers [27]. Another contributing factor might be the increased presence of household air pollution (HAP) in rural areas, a topic we will explore further. Additionally, compared with rural residents, urban residents have a significantly higher intake of fruits and vegetables [28]. These factors likely contribute to the urban-rural disparity in NPC mortality in China.

In this study, the crude and standardized NPC mortality rates in the four groups (urban males, urban females, rural males and rural females) from 2006 to 2020 exhibited a stable downward trend. Among these groups, urban females experienced the most substantial rate of decline, followed by urban males, while rural males showed the smallest decrease. This decline in mortality rates may be attributed to improvements in living conditions, medical care, and screening technology. In 2009, China launched a major health care reform, promising to provide all citizens with equal access to basic health care [29]. The government has invested substantial amounts of money to establish a universal primary health care system. Given the yet incompletely understood etiology of NPC, secondary prevention is highly important. China has carried out several screening programs in high-incidence areas. For instance, since 1986, 98,180 residents have participated in an NPC screening program targeting high-risk groups in Guangdong Province. The 5-year survival rate among screened participants reached 79.87%, which was significantly higher than the 58.43% rate observed among hospitalized cases during the same period [30].

From 2006 to 2020, among urban males, the most significant decline in NPC mortality was observed in the age group 35–39, while the 40–44 age groups exhibited the most prominent decrease in the other three groups. These findings showed the notable advancements in NPC prevention and treatment in young and middle-aged people in China. Among the age-period-cohort effects, the age effect coefficient exhibited the widest span, signifying its predominant influence on NPC mortality risk. The NPC mortality risk increased with age but plateaued in older age groups or even exhibited a slight decline, aligning with previous research findings [7]. Many studies on NPC prognosis have consistently reported higher overall survival rates in young patients [31]. This phenomenon may be attributed to lower immunity, increased comorbidities, and higher nutritional requirements in elderly individuals [32]. Reports have indicated that elderly individuals in China often fail to meet recommended dietary guidelines for fruit and vegetable consumption, with 85.2% and 49.6% of them falling short in fruit and vegetable intake, respectively, in 2015 [33]. Enhancing dietary education in elderly individuals could serve as an effective measure for NPC prevention and treatment.

In rural areas, there was an increase in the period effect in 2016–2020, signifying a rise in NPC mortality risk. Furthermore, the results revealed a widening gap in standardized mortality between urban and rural areas in recent years. This might be associated with HAP, primarily caused by solid fuel usage in household activities such as cooking and heating. Solid fuels such as coal and biomass are commonly prevalent in inadequately ventilated rural households. While China has witnessed a decrease in solid fuel consumption over the past decade, this shift is mainly attributed to urbanization rather than specific household control policies [34]. As of 2014, approximately 48% of rural households used solid fuels for cooking, 72% for heating, and over half of them lacked adequate ventilation, in contrast to less than 5% of urban households [35]. Indoor air in these rural households often contains levels of particulate matter and nitrogen dioxide that exceed the World Health Organization (WHO) recommendations and Chinese national standards. Both of these pollutants have been significantly linked to NPC risk [36]. Importantly, rural females are more frequently to be exposed to HAP from solid fuels usage in cooking and heating, resulting in heightened air pollution exposure [37]. This may elucidate the relatively modest reduction in standardized NPC mortality among rural females. However, the adverse effects of HAP extend far beyond NPC risk; research indicates that nearly 3 million premature deaths occur globally due to the use of solid fuels for cooking among 2.8 billion people [38]. Therefore, enhancing infrastructure and promoting energy upgrades in rural households is of paramount importance.

While China ratified the WHO Framework Convention on Tobacco Control in 2003, the tobacco control policies implemented in China have seen limited success in reducing the smoking rate. Notably, the prevalence of smoking has exhibited an alarming rise among adolescents of both genders, with a steady increase observed among young females [39]. Furthermore, the prevalence of secondhand smoke exposure in households with school-aged children remains unacceptably high [40]. Epidemiological investigations have demonstrated that individuals who commence smoking at an early age face an elevated risk of developing NPC [41]. This observation potentially elucidates the slower rate of increase or decline in risk coefficients among males and females within later-born cohorts in our study. With China’s sustained economic development, increasing disparities in education and income levels between urban and rural regions have become apparent, thereby giving rise to various challenges faced by homeschooled children. Adolescents residing in rural areas, characterized by lower levels of social connectedness and heightened emotional challenges, may turn to tobacco consumption as a coping mechanism to alleviate stress [42]. Recent trends have also indicated a surge in smoking prevalence, particularly among less educated males born after 1980, with the increase being notably pronounced in this subgroup compared to their highly educated counterparts [43]. This phenomenon may explain the observed increased risk coefficients among rural males within later-born cohorts. These findings emphasize the critical necessity to monitor and address smoking behaviors among young cohorts, with a specific focus on individuals with lower educational achievement. Additionally, several studies have reported that the rapid economic growth in China has translated into a widening cancer mortality gap between rural and urban residents, with rural populations experiencing a higher cancer mortality rate [44]. To mitigate this health disparity between urban and rural areas, it is imperative to bolster the development of medical infrastructure in rural regions and allocate increased educational resources to these areas.

### Limitations

The findings of this study have offered epidemiological evidence regarding NPC mortality in China from 2006 to 2020. Nonetheless, the study comes with certain limitations. Firstly, the utilized database lacks data concerning the NPC incidence rate, rendering it impossible to conduct a comprehensive analysis of the epidemic trends associated with NPC. Secondly, it’s important to acknowledge that the application of age-period-cohort analysis inherently entails ecological fallacies, which caution against generalizing the study’s results to individual-level interpretations.

## Conclusions

Overall, the study showed a consistent decrease in NPC mortality rates across China from 2006 to 2020, reflecting effective prevention and treatment strategies. However, there remained an urban-rural gap, with rural areas experiencing a rise in NPC mortality during 2016–2020. This can be attributed to poorer healthcare infrastructure, dietary practices, and increased indoor air pollution in rural regions.

### Electronic supplementary material

Below is the link to the electronic supplementary material.

Additional File 1: Table A. 1. The crude and standardized mortality rates of NPC by region and sex in China (1/100,000).

Additional File 2: Table A. 2. Effect coefficients of the NPC mortality of age, period, and cohort factors.

## Data Availability

All the data was sourced from the China Health Statistics Yearbook, which was publicly available at the website http://www.nhc.gov.cn/mohwsbwstjxxzx/new_index.shtml. If someone wants to request the data from this study, also can contact the corresponding author by email koucg@jlu.edu.cn.

## List of abbreviations

NPC: Nasopharyngeal carcinoma
APC: Annual percentage change
AAPC: Average annual percentage change
IE: Intrinsic estimator
EBV: Epstein–Barr virus
HAP: Household air pollution
WHO: World Health Organization
