# Supplementary material for: Long-term trends of nasopharyngeal carcinoma mortality in China from 2006 to 2020 by region and sex: an age-period-cohort analysis
Source: BMC Public Health. 2023 Oct 20;23:2057. doi: 10.1186/s12889-023-16892-1 (PMC10588046; doi:10.1186/s12889-023-16892-1)
Supplement: Supplementary file 1 — Additional File 1: Table A. 1. The crude and standardized mortality rates of NPC by region and sex in China (1/100,000). [file 12889_2023_16892_MOESM1_ESM.doc]

**Additional File 1**

**Table A. 1.** The crude and standardized mortality rates of NPC by region and sex in China (1/100,000)

| Year | Urban male | | Urban female | |
| --- | --- | --- | --- | --- |
|  | Standardized mortality | Crude mortality | Standardized mortality | Crude mortality |
| 2006 | 4.570 | 4.323 | 1.719 | 1.615 |
| 2007 | 5.372 | 5.176 | 1.817 | 1.739 |
| 2008 | 3.959 | 3.856 | 1.252 | 1.228 |
| 2009 | 3.669 | 3.665 | 1.262 | 1.236 |
| 2010 | 3.490 | 3.149 | 1.395 | 1.252 |
| 2011 | 4.183 | 3.870 | 1.404 | 1.290 |
| 2012 | 3.660 | 3.397 | 1.276 | 1.182 |
| 2013 | 3.069 | 2.854 | 0.981 | 0.936 |
| 2014 | 3.231 | 3.053 | 0.987 | 0.953 |
| 2015 | 3.089 | 2.980 | 0.984 | 0.961 |
| 2016 | 2.762 | 2.702 | 0.858 | 0.829 |
| 2017 | 2.879 | 2.852 | 0.971 | 0.950 |
| 2018 | 2.887 | 2.874 | 0.939 | 0.932 |
| 2019 | 2.699 | 2.702 | 0.831 | 0.828 |
| 2020 | 2.419 | 2.419 | 0.705 | 0.705 |

| Year | Rural male | | Rural female | |
| --- | --- | --- | --- | --- |
|  | Standardized mortality | Crude mortality | Standardized mortality | Crude mortality |
| 2006 | 6.982 | 6.070 | 2.404 | 1.957 |
| 2007 | 5.008 | 4.398 | 1.676 | 1.412 |
| 2008 | 4.261 | 3.803 | 1.469 | 1.304 |
| 2009 | 5.389 | 4.862 | 1.873 | 1.616 |
| 2010 | 5.882 | 5.013 | 2.007 | 1.660 |
| 2011 | 4.320 | 3.869 | 1.654 | 1.438 |
| 2012 | 4.123 | 3.727 | 1.508 | 1.389 |
| 2013 | 3.799 | 3.395 | 1.360 | 1.189 |
| 2014 | 4.046 | 3.715 | 1.344 | 1.202 |
| 2015 | 4.260 | 3.972 | 1.350 | 1.221 |
| 2016 | 4.228 | 4.028 | 1.364 | 1.273 |
| 2017 | 4.096 | 3.936 | 1.442 | 1.357 |
| 2018 | 4.439 | 4.320 | 1.449 | 1.384 |
| 2019 | 3.989 | 3.885 | 1.370 | 1.319 |
| 2020 | 3.787 | 3.787 | 1.327 | 1.328 |
