# Supplementary material for: Long-term trends of nasopharyngeal carcinoma mortality in China from 2006 to 2020 by region and sex: an age-period-cohort analysis
Source: BMC Public Health. 2023 Oct 20;23:2057. doi: 10.1186/s12889-023-16892-1 (PMC10588046; doi:10.1186/s12889-023-16892-1)
Supplement: Supplementary file 2 — Additional File 2: Table A. 2. Effect coefficients of the NPC mortality of age, period, and cohort factors. [file 12889_2023_16892_MOESM2_ESM.docx]

**Additional File 2**

**Table A. 2.** Effect coefficients of the NPC mortality of age, period, and cohort factors

| Factor | Urban male | | | Urban female | | |
| --- | --- | --- | --- | --- | --- | --- |
|  | Effect coefficient | 95%CI | P-value | Effect coefficient | 95%CI | P-value |
| Age |  |  |  |  |  |  |
| 30-34 | -1.432 | (-1.577, -1.286) | < 0.001 | -1.185 | (-1.411, -0.959) | < 0.001 |
| 35-39 | -0.838 | (-0.926, -0.750) | < 0.001 | -0.803 | (-0.951, -0.654) | < 0.001 |
| 40-44 | -0.357 | (-0.433, -0.281) | < 0.001 | -0.273 | (-0.398, -0.147) | < 0.001 |
| 45-49 | -0.128 | (-0.200, -0.055) | 0.001 | -0.260 | (-0.387, -0.132) | < 0.001 |
| 50-54 | 0.271 | (0.205, 0.338) | < 0.001 | 0.051 | (-0.067, 0.169) | 0.398 |
| 55-59 | 0.266 | (0.203, 0.329) | < 0.001 | 0.127 | (0.014, 0.240) | 0.028 |
| 60-64 | 0.465 | (0.407, 0.523) | < 0.001 | 0.386 | (0.286, 0.486) | < 0.001 |
| 65-69 | 0.635 | (0.580, 0.689) | < 0.001 | 0.512 | (0.420, 0.605) | < 0.001 |
| 70-74 | 0.585 | (0.525, 0.645) | < 0.001 | 0.701 | (0.605, 0.798) | < 0.001 |
| 75-79 | 0.532 | (0.450, 0.614) | < 0.001 | 0.743 | (0.615, 0.870) | < 0.001 |
| Period |  |  |  |  |  |  |
| 2006-2010 | 0.088 | (0.062, 0.114) | < 0.001 | 0.172 | (0.128, 0.217) | < 0.001 |
| 2011-2015 | 0.012 | (-0.013, 0.037) | 0.341 | -0.006 | (-0.050, 0.038) | 0.787 |
| 2016--2020 | -0.101 | (-0.126, -0.075) | < 0.001 | -0.166 | (-0.212, -0.121) | < 0.001 |
| Cohort |  |  |  |  |  |  |
| 1929-1933 | 0.691 | (0.576, 0.806) | < 0.001 | 0.499 | (0.316, 0.682) | < 0.001 |
| 1934-1938 | 0.455 | (0.377, 0.532) | < 0.001 | 0.485 | (0.364, 0.605) | < 0.001 |
| 1939-1943 | 0.371 | (0.310, 0.432) | < 0.001 | 0.427 | (0.330, 0.525) | < 0.001 |
| 1944-1948 | 0.364 | (0.304, 0.425) | < 0.001 | 0.335 | (0.232, 0.437) | < 0.001 |
| 1949-1953 | 0.282 | (0.218, 0.347) | < 0.001 | 0.299 | (0.187, 0.411) | < 0.001 |
| 1954-1958 | 0.203 | (0.133, 0.273) | < 0.001 | 0.203 | (0.079, 0.328) | 0.001 |
| 1959-1963 | 0.044 | (-0.033, 0.121) | 0.260 | 0.026 | (-0.112, 0.164) | 0.709 |
| 1964-1968 | -0.183 | (-0.262, -0.103) | < 0.001 | -0.033 | (-0.169, 0.103) | 0.632 |
| 1969-1973 | -0.293 | (-0.374, -0.211) | < 0.001 | -0.250 | (-0.390, -0.111) | < 0.001 |
| 1974-1978 | -0.588 | (-0.679, -0.497) | < 0.001 | -0.635 | (-0.788, -0.482) | < 0.001 |
| 1979-1983 | -0.723 | (-0.846, -0.599) | < 0.001 | -0.676 | (-0.881, -0.471) | < 0.001 |
| 1984-1988 | -0.625 | (-0.855, -0.394) | < 0.001 | -0.679 | (-1.052, -0.307) | < 0.001 |

| Factor | Rural male | | | Rural female | | |
| --- | --- | --- | --- | --- | --- | --- |
|  | Effect coefficient | 95%CI | P-value | Effect coefficient | 95%CI | P-value |
| Age |  |  |  |  |  |  |
| 30-34 | -1.720 | (-1.826, -1.613) | < 0.001 | -1.135 | (-1.273, -0.997) | < 0.001 |
| 35-39 | -0.891 | (-0.951, -0.830) | < 0.001 | -0.810 | (-0.903, -0.718) | < 0.001 |
| 40-44 | -0.143 | (-0.192, -0.094) | < 0.001 | -0.371 | (-0.450, -0.293) | < 0.001 |
| 45-49 | 0.052 | (0.005, 0.098) | 0.030 | -0.172 | (-0.250, -0.095) | < 0.001 |
| 50-54 | 0.415 | (0.373, 0.456) | < 0.001 | 0.148 | (0.077, 0.219) | < 0.001 |
| 55-59 | 0.460 | (0.422, 0.499) | < 0.001 | 0.325 | (0.258, 0.392) | < 0.001 |
| 60-64 | 0.515 | (0.481, 0.549) | < 0.001 | 0.453 | (0.394, 0.512) | < 0.001 |
| 65-69 | 0.505 | (0.474, 0.537) | < 0.001 | 0.474 | (0.419, 0.529) | < 0.001 |
| 70-74 | 0.428 | (0.392, 0.464) | < 0.001 | 0.587 | (0.529, 0.645) | < 0.001 |
| 75-79 | 0.379 | (0.329, 0.428) | < 0.001 | 0.502 | (0.423, 0.581) | < 0.001 |
| Period |  |  |  |  |  |  |
| 2006-2010 | 0.052 | (0.037, 0.068) | < 0.001 | 0.067 | (0.040, 0.094) | < 0.001 |
| 2011-2015 | -0.091 | (-0.107, -0.076) | < 0.001 | -0.072 | (-0.099, -0.045) | < 0.001 |
| 2016-2020 | 0.039 | (0.023, 0.055) | < 0.001 | 0.005 | (-0.023, 0.033) | 0.726 |
| Cohort |  |  |  |  |  |  |
| 1929-1933 | 0.684 | (0.615, 0.754) | < 0.001 | 0.695 | (0.587, 0.803) | < 0.001 |
| 1934-1938 | 0.608 | (0.562, 0.654) | < 0.001 | 0.540 | (0.467, 0.613) | < 0.001 |
| 1939-1943 | 0.440 | (0.404, 0.476) | < 0.001 | 0.420 | (0.362, 0.479) | < 0.001 |
| 1944-1948 | 0.290 | (0.253, 0.326) | < 0.001 | 0.255 | (0.195, 0.315) | < 0.001 |
| 1949-1953 | 0.222 | (0.184, 0.261) | < 0.001 | 0.165 | (0.099, 0.232) | < 0.001 |
| 1954-1958 | -0.025 | (-0.069, 0.018) | 0.254 | -0.043 | (-0.118, 0.032) | 0.262 |
| 1959-1963 | -0.272 | (-0.322, -0.222) | < 0.001 | -0.183 | (-0.268, -0.098) | < 0.001 |
| 1964-1968 | -0.188 | (-0.238, -0.138) | < 0.001 | -0.055 | (-0.138, 0.028) | 0.195 |
| 1969-1973 | -0.450 | (-0.505, -0.395) | < 0.001 | -0.294 | (-0.382, -0.206) | < 0.001 |
| 1974-1978 | -0.648 | (-0.712, -0.585) | < 0.001 | -0.534 | (-0.632, -0.435) | < 0.001 |
| 1979-1983 | -0.411 | (-0.496, -0.326) | < 0.001 | -0.410 | (-0.539, -0.281) | < 0.001 |
| 1984-1988 | -0.250 | (-0.418, -0.082) | 0.004 | -0.557 | (-0.796, -0.317) | < 0.001 |

CI: confidence interval.
